# Implementation of the Baveno Classification in Obstructive Sleep Apnea and Its Correlation with Symptoms of Anxiety and Depression

**DOI:** 10.3390/medicina59111938

**Published:** 2023-11-01

**Authors:** Romana Suša, Miloš Ratinac, Vojislav Ćupurdija, Ljiljana Novković, Mirjana Milojević-Ilić, Marina Petrović, Nebojša Igrutinović, Marko Vuleta, Ljiljana Timotijević, Olivera Kostić, Ivan Čekerevac

**Affiliations:** 1 Pulmonology Clinic, University Clinical Centre Kragujevac, 34000 Kragujevac, Serbia; romanasusa@gmail.com (R.S.); vojacup@gmail.com (V.Ć.); shone31094@gmail.com (N.I.); icekerevac@gmail.com (I.Č.); 2 Faculty of Medical Sciences, University of Kragujevac, 34000 Kragujevac, Serbia; 3 Department of Internal Medicine, Faculty of Medical Sciences, University of Kragujevac, 34000 Kragujevac, Serbia; 4 Department of Cardiology, Clinical Hospital Center Dr Dragisa Misovic, 11000 Beograd, Serbia; 5 Institute for Pulmonary Diseases Beograd, 11000 Beograd, Serbia; 6 Department of Pharmacy, Faculty of Medical Sciences, University of Kragujevac, 34000 Kragujevac, Serbia; 7 Center for Harm Reduction of Biological and Chemical Hazards, Faculty of Medical Sciences, University of Kragujevac, 34000 Kragujevac, Serbia

**Keywords:** obstructive sleep apnea, AHI, Baveno classification, anxiety, depression

## Abstract

*Background and Objectives:* The Baveno classification represents a new approach to the assessment of the severity of OSA (Obstructive sleep apnea), which takes significant comorbidities into account: atrial fibrillation, arterial hypertension, heart failure, stroke, diabetes mellitus, and OSA symptoms expressed through the Epworth sleepiness scale (ESS). The authors believe that the Baveno classification facilitates a better stratification of patients with OSA and can be a good guide for deciding on the therapeutic approach and clinical monitoring of patients with OSA, compared to the AHI (apnea-hypopnea index) itself. The aim of this paper is to confirm the advantage of applying the Baveno classification to the evaluation of symptoms of anxiety and depression in the OSA patients compared to the application of the AHI as a single parameter. *Materials and Methods:* This research represents an observational retrospective study that was performed at the Pulmonology Clinic of the University Clinical Center in Kragujevac, Serbia. The study sample included 104 patients with diagnosed OSA. Patients were divided into four categories retrogradely according to the Baveno classification (A, B, C, and D). Statistical data processing was performed using the IBM SPSS Statistics version 25.0 program. *Results:* In our study, we proved that the Baveno classification is better at predicting the depressive disorder in OSA patients compared to the AHI itself, according to abnormal BDI-II (Beck Depression Inventory) score (value greater than ten) and HADS-D (Hospital anxiety and depression) scale (value greater than eight). The average AHI in the entire group of examined patients was 44.3 ± 19.8, while in category A the average AHI was 25.2 ± 10, in category B, 53.4 ± 20.6; in category C, 38.2 ± 18.5; and in category D, 48.1 ± 19.2. In the total sample, AHI did not correlate with the depressive episodes, but individually, the highest frequency of the depressive symptoms was precisely in the categories with the highest AHI (group D and B), where more than half of the subjects had an abnormal score. The frequency of the anxiety disorder (HADS-A) between the analyzed groups did not differ significantly, although the largest number of patients with significant anxiety were in category B, according to the Baveno classification. *Conclusions:* We proved that the Baveno classification is applicable in real life, and it is better at evaluating anxiety and depression using questionnaires and can identify new patients who need CPAP therapy, independently of other OSAS symptoms, primarily daytime sleepiness.

## 1. Introduction

Obstructive sleep apnea (OSA) is the most common breathing disorder occurring during sleep, which is characterized by episodes of partial (hypopnea) or complete (apnea) collapse of the upper airways followed by blood oxygen desaturation and sudden awakening from sleep [1]. OSA affects between 9–13% of the general population, significantly increasing the overall morbidity and mortality rate [2]. Besides OSA’s influence on mortality and mobility, it is important to underline the influence of this disease on financial costs that can affect an individual and the society as well. The negative influence of OSA on the patient’s quality of life is properly documented by numerous studies. Except the physical health itself, OSA has a great impact on mental health and emotional status, and several studies confirmed that OSA may even worsen the bed partner’s quality of life due to snoring during the night and consequently decrease the energy and vitality of the bed partner [2,3,4]. The disease rate is slightly higher in the male population and has not changed significantly in the previous decade [5]. The diagnosis is confirmed by an overnight sleep study or polysomnography, and the severity of the disease is expressed by the apnea-hypopnea index (AHI). This index was used for decades to diagnose and stratify the severity of OSA, however, despite its widespread use, research has shown that the AHI has a poor correlation with the OSA symptoms, morbidity and mortality, in other words, it represents a simplification of a complex and diverse process [6]. It equates episodes of apnea and hypopnea, considering them as equal events, and does not take into account their duration or the level of desaturation [7]. Also, an additional characteristic of this index is that it does not adequately correlate with the symptom severity, as patients with a high AHI may have mild symptoms and vice versa, which may be explained by differences in individual sensitivity to the systemic effects of OSA [8]. On the other hand, OSA is associated with systemic comorbidities (including cardiovascular, metabolic, neuropsychiatric, out of which anxiety and depression stand out) in a two-way connection, more precisely, OSA is imposed as an independent risk factor for numerous comorbidities, but there is evidence that some of these comorbidities can predispose to the development of OSA [9].

In addition, it is important to highlight that obstructive sleep apnea is more than sleepiness because it brings a number of specific and nonspecific symptoms, which can influence on the patient’s quality of life. According to available data, untreated and undiagnosed OSA leads to a decrease in work productivity, increased number of accidents at work or in traffic, and it can increase health system utilization and consequently create greater costs for the patient and health system as well [3,4].

Risk factors for OSA are divided into changeable and non-changeable. Non-changeable include male sex, age, and race. Changeable factors include obesity, the use of drugs that may cause muscle relaxation (opiates, benzodiazepines, and alcohol), endocrinological disorders (hypothyroidism), smoking, and nasal obstruction. Therefore, knowing the risk factors for obstructive sleep apnea is of great importance to direct diagnostic attention to the patients with the highest risk.

Intermittent hypoxemia is a contributing factor in the pathogenesis of comorbidities associated with OSA. Intermittent hypoxemia initiates oxidative stress with increased production of reactive oxygen species and angiogenesis, increased sympathetic activation, and systemic vascular inflammation with endothelial dysfunction [6,7].

The link between depression and OSA is complicated by the fact that both diseases have a significant number of overlapping symptoms. A literature search shows that the prevalence of the depressive and anxiety symptoms in patients with OSA varies from 15.5% to 35% and 14.4% to 32% retrospectively [9,10,11].

The Baveno classification represents a new approach to the assessment of the severity of OSA, which takes into account significant comorbidities: atrial fibrillation (paroxysmal, permanent, persistent, and recurrent after drug therapy or electro-conversion), arterial hypertension (therapy-resistant or uncontrolled), heart failure (NYHA II-IV, according to classification of the New York Hearth Association), stroke, diabetes mellitus, and OSA symptoms expressed through the Epworth sleepiness scale [12]. According to the Baveno classification, there are four groups of patients: group A with minor symptoms and comorbidities, group B with severe symptoms and minor comorbidities, group C with minor symptoms and severe comorbidities, and group D with severe symptoms and comorbidities. The symptoms of obstructive sleep apnea (OSA) include excessive daytime sleepiness, snoring, episodes of stopped breathing during sleep, night awakenings, dry mouth or sore throat in the morning, headache, “brain fog”, mood changes, depression, anxiety, arterial hypertension, and more [7,8]. The authors believe that this classification facilitates a better stratification of patients with OSA and can be a good guide for deciding on the therapeutic approach and clinical monitoring of patients with OSA, compared to the AHI itself [9]. According to this classification, patients can be classified into one of four groups: A, B, C, or D depending on the severity of the OSA symptoms and the presence and severity of comorbidities [12]. A potential shortcoming of the Baveno classification is that there are no long-term prospective studies that would affirm the clinical significance of this classification.

The aim of this paper is: to confirm the advantage of applying the Baveno classification in the evaluation of symptoms of anxiety and depression in OSA patients compared to the application of the AHI as a single parameter.

## 2. Materials and Methods

This research represents an observational retrospective study that was performed at the Pulmonology Clinic of the University Clinical Center in Kragujevac, Serbia. Study included patients over 18 years of age who were diagnosed with OSA (with an AHI ≥ 15), based on a sleep study (polygraphy or polysomnography) performed with Philips ALICE PdX device. The diagnoses were made in the period from July 2018 to November 2019 and in 2022 (after the clinic’s exit from the COVID-19 epidemic system). A total of 104 patients were included in this study, and they all provided written informed consent. Patients with previous pulmonary diseases (chronic obstructive pulmonary disease, chronic respiratory insufficiency, severe asthma, pulmonary fibrosis) or with previously diagnosed psychiatric diseases for which adequate therapy was initiated (anxiety, depression, psychosis, personality disorder), as well as patients with mild OSA (AHI < 15) were excluded. The study was conducted in the accordance with the Declaration of Helsinki and approved by the Ethics Committee of the University Clinical Center in Kragujevac no. 01/18-2488. All patients read and signed informed consent before they were enrolled in the study. The informed consent included all data about study protocol and information about confidentiality of patient personal and clinical information.

Measurements of vital parameters were performed on all patients on admission: blood pressure, oxygen saturation, neck circumference (NC), body weight, and height. Body mass index (BMI) was calculated for all patients. Based on the BMI value, patients were divided into normally nourished and obese (BMI ≥ 30).

All included patients filled out the Serbian version of the Epworth Sleepiness Scale (ESS) before undergoing the sleep study. This test is a list of eight situations in which the patient rates her/his tendency to become sleepy on a scale 0–3. The ESS score can range from 0 to 24 [13]. Also, all included patients filled out the Stop Bang scale (SBS). The following parameters were scored: snoring, tiredness, observed apnea, high blood pressure, body mass index (BMI), age, neck circumference (NC), and male gender. Based on this scale, patients can have a low or high risk for OSA. Two psychiatric assessment instruments, previously used in earlier studies in Serbia and worldwide, were also used in this study: Beck Depression Inventory (BDI-II) [14,15] and Hospital anxiety and depression scale (HADS) [16]. Also, patients were interviewed about associated diseases. The BDI-II is a 21-item questionnaire with responses ranging from 0 (no symptoms) to 3 (severe symptoms). The results were interpreted as follows: 0–9 normal score; 10–18 mild to moderate depression; 19–29 moderate to severe depression; 30–60 severe depression. The HADS is a questionnaire consisting of 14 items (7 items each for anxiety and depression subscales); scoring for each scale ranges from 0 (absence of symptoms) to 3 (severe symptoms). A subscale score > 8 indicates an anxiety or depressive disorder.

Patients were retrogradely divided into 4 categories according to the Baveno classification, based on ESS values and data on important comorbidities obtained anamnestically, from hospital discharge reports, and the health information system: group A (ESS < 11, patient without comorbidities or they are well controlled); group B (ESS ≥ 11, patient without comorbidities or they are well controlled); group C (ESS < 11, patient with emphasized comorbidities that are poorly controlled); group D (ESS ≥ 11, patient with emphasized comorbidities that are poorly controlled) [12].

## 3. Statistical Analysis

The data used were defined by descriptive statistics and analyzed using adequate statistical methods. Depending on the variable type, the results were expressed as numbers and percentages (categorical variables) and mean ± SD (continuous variable). Normality of continuous data was confirmed by the Shapiro–Wilk normality test. The difference in the average values of continuous data by groups was tested by Analysis of variance (ANOVA) test. The difference in the frequency of categorical data was tested with the chi square (χ^2^) test. Statistical data processing was performed using the IBM SPSS Statistics version 25.0 program. For testing of categorical data of two or more observations, the χ² test was used in the form of contingency tables, and in the case of testing the difference in the frequency of one observation and in the case of testing the difference in the frequency of one observation, the χ^2^ test was used in the form of an agreement test. The significance of the association between AHI and Beck’s scale and ESS was tested with the Pearson correlation coefficient. The results were considered statistically significant if the significance (*p* value) was less than or equal to 0.05.

## 4. Results

Of a total of 104 patients with AHI ≥ 15 who were included in this study, according to the Baveno classification, 5.8% of patients were classified into group A, 10.6% of patients were classified into group B, 29.8% of patients into group C, and 53.8% of patients into group D. The average AHI value in the entire group of examined patients was 44.3 ± 19.8, while in category A, the average AHI value was 25.2 ± 10, in category B, 53.4 ± 20.6; in category C, 38.2 ± 18.5; and in category D, 48.1 ± 19.2. In each of the Baveno categories, the frequency of males was significantly higher, ranging from 66.7% in group B to 77.4% in group C (χ^2^ = 41.08, df = 3, *p* < 0.01). No statistically significant difference in age was observed between the categories. Of the total number of patients who were diagnosed with OSA during the study period, 74% of them were male (χ^2^ = 24.04, df = 1, *p* < 0.01).

Of the total number of the included patients, 79.8% of patients had significant arterial hypertension (HTA). According to the Baveno classification, there were 1.9% of patients in category A, 1% of patients in category B, 26% of patients in category C, and 51% of patients in category D with significant arterial hypertension. The frequency of hypertension is significantly different between the Baveno groups (χ^2^ = 50.83, df = 3, *p* < 0.05). Diabetes mellitus (DM) was present in 37.5% of patients, with no diabetics in categories A and B, while in category C there were 9.6%, and in category D, 27.9% of diabetics. The frequency of DM is different in the analyzed groups (χ^2^ = 15.44, df = 3, *p* < 0.05). Atrial fibrillation and significant heart failure were not considered due to the small number of patients who had these comorbidities. The largest number of the obese patients was in group D (82.1%), and the smallest in group A (33.3%), (χ^2^ = 8.09, df = 3, *p* < 0.05) (Table 1).

The average value of ESS score was 12.4 ± 4.9, and 66.6% of patients had ESS ≥ 11. A statistically significant positive, moderately strong correlation between AHI and ESS was observed (r = 0.34, *p* < 0.01). The average value of ESS significantly differs in the given groups (F = 39.94, df = 3, *p* < 0.01). Post hoc testing revealed a significant difference in average ESS values between groups A and B, A and D, then B and C, and finally, C and D groups.

**Figure 1:**
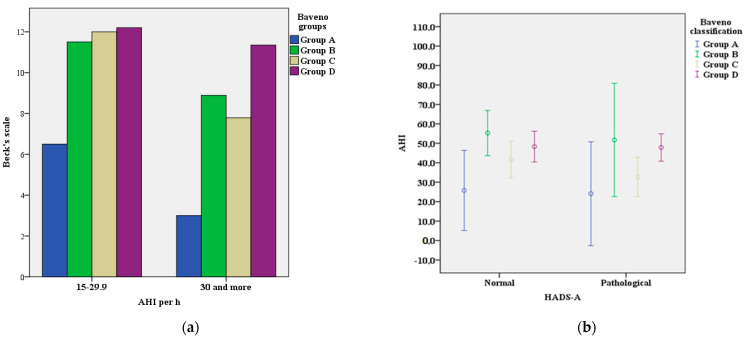
(**a**) Correlation between AHI and Beck depression scale (Beck’s scale) in relation to Baveno groups. (**b**) Correlation between AHI and HADS-A in relation to Baveno groups.

In our sample, 55 patients (42.9%) had abnormal BDI-II scores (value greater than 10). BDI-II scores differ significantly between the categories, from 16.7% in category A to 58.5% in category D (χ^2^ = 24.4, df = 3, *p* < 0.01) (Table 2). In the total sample, the correlation between AHI and Beck’s scale does not reach the threshold of statistical significance (r = 0.025, *p* > 0.05) (Figure 1a).

According to the Hospital Anxiety and Depression Scale (HADS), two patients (33.3%) in category A, six patients (54.5%) in category B, fifteen patients (48.4%) in category C, and thirty-six patients (67.9%) in category D had an abnormal finding (score > 8), (χ^2^ = 19.01, df = 3, *p* < 0.01). However, significant difference was observed in the frequency of normal and pathological HADS-D (χ^2^ = 4.88, df = 3, *p* < 0.01), but it was not observed between normal and pathological HADS-A (χ^2^ = 1.06, df = 3, *p* > 0.05) and the Baveno classification groups. The frequency of the anxiety disorder (HADS-A) in the analyzed groups did not differ significantly, although the largest number of patients with significant anxiety was in category B (χ^2^ = 5.72, df = 3, *p* > 0.05), (Table 2; Figure 1b). Furthermore, the representation of patients with the anxiety disorder (=43) in the Baveno groups shows that the largest number of them was in group D, 53.5% (*n* = 23) and group C, 27.9% (*n* = 12) and the smallest in group A 4.7% (*n* = 2). (χ^2^ = 23.33, df = 3, *p* < 0.01).

The analysis of patients who had the depressive disorder (*n* = 59) shows that the largest number was in the Baveno group D, 61% (*n* = 36) and group C, 25.4% (*n* = 15), and the smallest in group A 3.4% (*n* = 2), (χ^2^ = 46.83, df = 3, *p* < 0.01).

## 5. Discussion

According to the available literature evidence, it is clear that there is a positive relation between OSA and psychological disorders such as depression or anxiety, and that these conditions are some of the OSA comorbidities. These symptoms are explained by the influence of OSA on the structures of the central nervous system by diminishing the volume of the hippocampal mass and affecting changes in the white matter. At neurochemical level, this produces disturbances in serotonin level, which contribute to depression. Even if these hormonal changes were previously described, closer relationship between OSA and depression/anxiety remains relatively unknown and still is in focus of a number of different investigations. Also, OSA and mental disorders can contribute to lower quality of life of patients by increased rate of alcohol, tobacco, or drug abuse, which may develop other health complications. These results clearly indicate the need for cooperation between pneumonologists and psychiatrists, as the result of cooperative treatment will provide the best healthcare for the patient. In contrast to this statement, there is also a large body of evidence that the correlation between psychological disease and the OSA level is not sufficiently explained, and conduction of larger clinical studies on this topic would be useful for further guidelines [3,4].

The results of our work support previous results, where it was observed that risk factors such as male sex, age (over 40), neck circumference > 40 cm, and obesity reveal individuals with a higher risk of developing OSA, as well as developing a more severe form of the disease [2,5]. The proportion of patients whose neck circumference was >40 cm in our study was 79.4%. (x^2^ = 35.29, df = 1, *p* < 0.01). Differences in distribution of patients among different Baveno groups were noted in relation to gender, neck circumference, and obesity. The mean age of the patients included in this study was 53.5 ± 12.1 and we did not prove a statistically significant difference between the Baveno groups (Table 1).

In our study, we have shown that the Baveno classification proved to be better in predicting the depressive disorder in OSA patients compared to the AHI itself, according to abnormal BDI-II scores (value greater than 10), and HADS-D (value greater than 8). We also found that according to the Baveno classification, the largest number of patients with significant anxiety was in category B. Symptoms of depression were assessed using two questionnaires, BDI-II and HADS-D, and the scores of both questionnaires differed significantly between the categories of the Baveno classification. In the total sample, AHI did not correlate with the depressive episodes, but individually, the highest frequency of the depressive symptoms was precisely in the categories with the highest AHI (groups D and B), where more than half of the subjects had an abnormal questionnaire score. The most depressed patients were those who were the most burdened by OSA symptoms and comorbidities (group D patients). When anxiety was analyzed, assessed by using the HADS-A questionnaire, it did not differ significantly among the Baveno categories of subjects, but considered individually, the most emphasized anxiety disorders were observed in group B, and then group D.

Our research shows a significant difference in the AHI index between the categories of patients in the Baveno classification. The lowest AHI was in group A, and the highest AHI scores were in group B and group D. This distribution was also proven in a paper by a group of authors which included 20 European countries and over 14.000 patients (10). A study conducted in Portugal on a sample of 91 patients did not show a significant difference in the AHI index between the distribution of patients by the Baveno groups. Although the authors of this study did not prove a relationship between the AHI index and comorbidities, a relatively higher AHI value was found in group D [17].

The ESS was the highest in the group of patients with emphasized OSA symptoms (B, D). A medium-strong (r = 0.34) positive correlation between the ESS and the AHI was found, which was also shown in other studies [18,19,20,21], where the AHI correlated poorly with the ESS values, especially in the female population [22]. Data from the European ESADA registry showed that individual polysomnographic parameters correlated poorly with symptoms and showed no clinically relevant difference between the Baveno groups (A + C vs. B + D) [23].

Reviewing the available literature, we found that symptoms of anxiety and depression in patients with OSA correlated negatively with the severity of the disease [11,18,19,24,25,26]. One of the few studies that proved the opposite is an Australian study involving 426 patients where the authors proved that the prevalence of the depressive symptoms was positively associated with the severity of OSA [18,26]. The frequency of the anxiety and depression disorders varied from study to study, sample size, and scales used, and ranged from 15.5% to 35% and 14.4% to 32% retrospectively [9,10,11]. One of the first and largest studies to analyze the risk of developing affective disorders among individuals with OSA was a study conducted on South Korean patients. In this study, the authors proved that patients with OSA had a risk of developing affective disorders (depression and anxiety) that was 2.04 times higher than the risk in patients without OSA. The follow-up period of these patients was nine years, and this was a cohort study using a nationwide population-based data set. Female patients were significantly more likely to develop depression and anxiety compared to male patients [27]. This leaves room for further research.

On the other hand, patients who have anxiety are 2.60 times more likely to develop OSA than those without this disease. Also, in another study, they showed that depression is associated with an increased risk of OSA after excluding patients older than 60 years [28].

Our findings are not in accordance with the results of previous studies in which symptoms of depression and anxiety negatively correlated with the severity of OSA [10,11,18,19].

Improvement of OSA with the CPAP therapy reduced the severity of the depression and anxiety symptoms [20], which indicates that the anxiety and depression symptoms may be the new criteria to be used as a parameter for initiation of CPAP therapy. The Baveno classification, as a multicomponent system, better assesses the need for the introduction of the CPAP therapy, without relying solely on daytime sleepiness as the guiding criterion. The main limitations of our study were that the sample on which we conducted our research was small, and other comorbidities important for Baveno classification of OSA severity, such as heart failure, heart rhythm disorders, and cerebrovascular diseases, were not included due to small number of cases presented in the observed sample. Another limiting factor of our research was the methodological setting of the study in terms of the inclusion criteria, as patients who had a mild form of OSA (AHI 5-15) were excluded from the study.

## 6. Conclusions

A higher frequency of the depressive disorder and anxiety was observed in patients burdened with OSA symptoms and comorbidities. We have shown that the Baveno classification is applicable in real life, better evaluates anxiety and depression using a questionnaire, and can indicate new patients who need CPAP therapy, independently of other OSAS symptoms, primarily daytime sleepiness.

## Figures and Tables

**Table 1:** Basic characteristic of the patients according to the Baveno classification.

Variables	TOTAL	Group A	Group B	Group C	Group D	*p*
	(*n*, %)	(*n*, %)	(*n*, %)	(*n*, %)	(*n*, %)	
Sex, male	77 (74)	4 (66.7)	9 (81.8)	24 (77.4)	40 (71.4)	<0.01
Age ($\bar{x}$ ± sd)	53.5 ± 12.1	47.8 ± 13.4	47.5 ± 13.1	54.5 ± 13.1	54.7 ± 10.9	>0.05
NC > 40 cm	81 (79.4)	4 (66.7)	8 (80)	23 (76.7)	46 (82.1)	<0.01
Obesity	79 (76)	2 (33.3)	7 (63.6)	24 (77.4)	46 (82.1)	<0.05
Hypertension	83 (79.8)	2 (1.9)	1 (1)	27 (26)	53 (51)	<0.05
Diabetes	39 (37.5)	/	/	10 (9.6)	29 (27.9)	<0.05

NC—neck circumference.

**Table 2:** Depression and anxiety scales, AHI and ESS in relation to Baveno groups.

Scale	Group A	Group B	Group C	Group D	*p*
AHI ($\bar{x}$ ± sd)	25.22 ± 10.15	53.36 ± 20.58	38.17 ± 18.51	48.05 ± 19.22	<0.05
ESS ($\bar{x}$ ± sd)	5.67 ± 4.23	14.91 ± 2.07	7.97 ± 2.92	15.05 ± 3.65	<0.01
SBS ($\bar{x}$ ± sd)	5 ± 1.27	5.64 ± 1.12	6.13 ± 1.18	6.48 ± 0.87	<0.05
BDI-II * (*n*, %)	1 (16.7)	6 (54.5)	14 (45.2)	31 (58.5)	<0.01
HADS-D * (*n*, %)	2 (33.3)	6 (54.5)	15 (48.4)	36 (67.9)	<0.01
HADS-A * (*n*, %)	2 (33.3)	6 (54.5)	12 (38.7)	23 (43.4)	>0.05

AHI—apnea-hypopnea index; BDI-II—Beck depression inventory; HADS-D—Hospital anxiety and depression scale—depression; HADS-A—Hospital anxiety and depression scale—anxiety; SBS—Stop Bang questionnaire; ESS—The Epworth Sleepiness Scale * pathological finding.

## Data Availability

The datasets used and analyzed during this study are available from the corresponding author upon reasonable request. The data were not publicly available because of ethical considerations.
